# Molecular Characterization and Response of Prolyl Hydroxylase Domain (PHD) Genes to Hypoxia Stress in *Hypophthalmichthys molitrix*

**DOI:** 10.3390/ani12020131

**Published:** 2022-01-06

**Authors:** Xiaohui Li, Meidong Zhang, Chen Ling, Hang Sha, Guiwei Zou, Hongwei Liang

**Affiliations:** 1 Yangtze River Fisheries Research Institute, Chinese Academy of Fishery Sciences, Wuhan 430223, China; lixiaohui@yfi.ac.cn (X.L.); zhangmeidong@yfi.ac.cn (M.Z.); lingchen@yfi.ac.cn (C.L.); sh1812@yfi.ac.cn (H.S.); 2 Key Laboratory of Aquatic Genomics, Ministry of Agriculture and Rural Affairs, Yangtze River Fisheries Research Institute, Chinese Academy of Fishery Science, Wuhan 430223, China; 3 State Key Laboratory of Developmental Biology of Feshwater Fish, College of Life Sciences, Hunan Normal University, Changsha 410081, China

**Keywords:** PHD, *hypophthalmichthys molitrix*, RACE, hypoxic stress, gene expression

## Abstract

**Simple Summary:**

Hypoxia is a common challenge for aquatic organisms, and prolyl hydroxylase domain (PHD) proteins play important roles in hypoxic adaptation by regulating the stability of the hypoxia-inducible factor 1 alpha subunit (HIF-1α). In this study, the full-length cDNAs of three PHD genes were obtained from *Hypophthalmichthys molitrix,* which is an important freshwater fish and sensitive to low oxygen tension. The amino acid sequence analysis and phylogenetic analysis of PHDs were performed among various species. Furthermore, the expression patterns and the transcriptional responses of *H. molitrix* PHD genes to acute hypoxia, continued hypoxia, and reoxygenation were explored in different tissues. Our study preliminarily explored the physiological regulation functions of PHD genes at the transcriptional level when addressing the hypoxic challenge and provided a foundation for future systematic explorations of the molecular mechanisms underlying hypoxia adaptation in silver carp.

**Abstract:**

As an economically and ecologically important freshwater fish, silver carp (*Hypophthalmichthys molitrix*) is sensitive to low oxygen tension. Prolyl hydroxylase domain (PHD) proteins are critical regulators of adaptive responses to hypoxia for their function of regulating the hypoxia inducible factor-1 alpha subunit (HIF-1α) stability via hydroxylation reaction. In the present study, three PHD genes were cloned from *H. molitrix* by rapid amplification of cDNA ends (RACE). The total length of *HmPHD1*, *HmPHD2*, and *HmPHD3* were 2981, 1954, and 1847 base pair (bp), and contained 1449, 1080, and 738 bp open reading frames (ORFs) that encoded 482, 359, and 245 amino acids (aa), respectively. Amino acid sequence analysis showed that HmPHD1, HmPHD2, and HmPHD3 had the conserved prolyl 4-hydroxylase alpha subunit homolog domains at their C-termini. Meanwhile, the evaluation of phylogeny revealed PHD2 and PHD3 of *H. molitrix* were more closely related as they belonged to sister clades, whereas the clade of PHD1 was relatively distant from these two. The transcripts of PHD genes are ubiquitously distributed in *H. molitrix* tissues, with the highest expressional level of *HmPHD1* and *HmPHD3* in liver, and *HmPHD2* in muscle. After acute hypoxic treatment for 0.5 h, PHD genes of *H. molitrix* were induced mainly in liver and brain, and different from *HmPHD1* and *HmPHD2*, the expression of *HmPHD3* showed no overt tissue specificity. Furthermore, under continued hypoxic condition, PHD genes exhibited an obviously rapid but gradually attenuated response from 3 h to 24 h, and upon reoxygenation, the transcriptional expression of PHD genes showed a decreasing trend in most of the tissues. These results indicate that the PHD genes of *H. molitrix* are involved in the early response to hypoxic stress, and they show tissue-specific transcript expression when performing physiological regulation functions. This study is of great relevance for advancing our understanding of how PHD genes are regulated when addressing the hypoxic challenge and provides a reference for the subsequent research of the molecular mechanisms underlying hypoxia adaptation in silver carp.

## 1. Introduction

Prolyl hydroxylase domain (PHD) proteins are oxygen-dependent enzymes, belonging to Fe (II) and 2-oxoglutarate-dependent dioxygenase family, which can regulate processes such as erythrocyte formation, angiogenesis, and heart development [1]. This family has three members, including PHD1, PHD2, and PHD3. PHDs are evolutionarily well-conserved proteins and share a similar C-terminal catalytic domain [2]. However, in addition to the typical C-terminal catalytic domain, PHD2 comprises a N-terminal myeloid (Myeloid, Nervy, DEAF-1) zinc finger structure (ZF-MYND), whereas PHD1 and PHD3 have lost this domain during the process of evolution [3]. PHDs were first found in *Caenorhabditis elegans* and named egl-9 family hypoxia-inducible factor 2, 1, 3 (EGLN2, 1, 3) [4], and then named as hypoxia inducible factor prolyl hydroxylase (HPH3, 2, 1) because they can hydroxylate the Pro402/564 site in the oxygen-dependent degradation domain (ODDD) of the hypoxia inducible factor-1 alpha (HIF-1α) [5].

Oxygen homeostasis is crucial for development, survival, and normal function of all animals [6,7]. Hypoxia can cause stress responses and even damage to organisms. In organisms, cells regulate some genes and proteins to adapt to hypoxia stress through oxygen receptors and signal transduction pathways [8]. HIF-1 signaling pathway is the most studied for its important regulatory effects in reducing oxygen consumption and increasing oxygen delivery [9], and in this pathway, the role of prolyl hydroxylase domain (PHDs) is particularly important for their function of regulating the hypoxia-inducible factors (HIFs) stability [10,11].

PHDs are important oxygen-sensing molecular switches in cells, and oxygen is necessary for PHDs catalytic activity [12]. Under normoxia condition, the PHDs hydroxylate the proline residues (Pro-402/564) of HIF-1α. Subsequently, the hydroxylated HIF-1α was recognized by von Hippel-Lindau tumor suppressor protein (VHL), combined to form a complex, and degraded by the E3 ubiquitin-degrading enzyme system mediated by the tumor suppressor protein [13]. However, under hypoxia, the catalytic activity of PHDs is inhibited [14]. Therefore, HIF-1α cannot be degraded by the E3 ubiquitin-degrading enzyme system, resulting in the accumulation of HIF-1α [15]. HIF-1α then binds to hypoxia inducible factor-1 beta (HIF-1β) in the nucleus to form a heterodimer, which could bind to its downstream hypoxia response element (HRE) to regulate gene expression, including oxygen sensor mobilization, oxygen transport, blood vessel, and erythropoietin production [16,17,18]. Therefore, as upstream regulatory genes of HIFs, PHDs have been confirmed to be critical mediators for the adaptive response to hypoxia [19,20].

To date, there have been fewer reports on the molecular and physiological studies of fish PHD genes. *PHD2* was cloned and identified from *Megalobrama amblycephala* [21] and *Sillago sihama* [22], respectively, and the results showed that *the* expression patterns of *PHD2* were different in these two species under hypoxic conditions. Moreover, the *MaPHD1* and *MaPHD3* were also cloned and identified [23,24]. The *MaPHD1* encodes two protein isoforms generated by alternative initiation, and both isoforms were distributed in the nucleus, therein the senior one promoted cell proliferation [23]. *MaPHD3* has two transcripts generated by alternative splicing, and both transcripts were up-regulated under hypoxia. Further evidence is needed to determine whether PHDs play different regulatory roles under hypoxia in different fish species [24]. *Hypophthalmichthys molitrix*, one of the most important aquaculture fish species in China, shows poor environmental hypoxia tolerance, making it hard to adapt to the hypoxic environment [25]. Therefore, it would be more meaningful for us to study the regulation functions of PHD genes in this highly hypoxia-sensitive fish.

In the present study, we aimed to analyze the expression patterns of PHD genes in different tissues and explore the transcriptional responses of PHD genes to acute hypoxia, continued hypoxia, and reoxygenation. These results will be of great relevance for advancing our understanding of how PHD genes perform physiological regulation functions when addressing the hypoxic challenge and provide a reference for the subsequent research of the molecular mechanisms underlying hypoxia adaptation in silver carp.

## 2. Materials and Methods

### 2.1. Experimental Fish

All silver carp used in this study were cultured in the Experimental Farm of Yangtze River Fisheries Research Institute, Chinese Academy of Fishery Sciences (YFI). Fish (100 ± 10 g) were stocked in tanks and allowed to acclimate for 7 days before starting the trail. The tanks were connected to an aerator to keep dissolved oxygen (DO; 6.5 ± 0.3 mg/L) stable.

### 2.2. Exposure and Tissue Collection

Silver carp were randomly selected from the acclimated fish for the experiment. Under a constant temperature (27.0 ± 0.5 °C), the normoxic condition (DO = 6.5 ± 0.3 mg/L) was maintained by inputting an amount of air, whereas the hypoxic conditions was created by rapidly filling nitrogen into the water. At the same time, the DO was monitored continuously using a water quality analyzer (HQ30D, Hach, Loveland, CO, USA).

The whole test process is inclusive of two parts, including the acute hypoxia experiment, and the continuous hypoxia-reoxygenation experiment. For acute hypoxia stress, silver carp were exposed to water at DO values of 2.5 mg/L (AH-2.5), 1.5 mg/L (AH-1.5), 0.5 mg/L (AH-0.5), and 0.25 mg/L (AH-0.25) for 0.5 h, respectively. For the continuous hypoxia-reoxygenation experiment, silver carp were first exposed to water at DO values of 1.5 ± 0.3 mg/L for 3 h (CH-3), 6 h (CH-6), 12 h (CH-12), and 24 h (CH-24), respectively, and after 24 h continuous hypoxia treatment, silver carp were secondly subjected to normoxic condition (DO = 6.5 ± 0.3 mg/L) to reoxygenate for 3 h (R-3), 6 h (R-6), 12 h (R-12), and 24 h (R-24). Before the start of the hypoxia experiment, fifteen fish (three replicates, each with 5 fish) were randomly selected from the acclimated fish as the control group (CK). After the exposure, fifteen fish were sampled at each time point (three replicates, each with 5 fish) during the acute hypoxia experiment, and continuous hypoxia-reoxygenation experiment. All fish were anaesthetized with a 0.05% solution of MS-222 before sampling. In the control group, tissue samples of liver, brain, heart, gill, muscle, intestine, kidney, and spleen were collected. In the experimental groups, tissue samples of liver, brain, heart, gill, and muscle were collected. The tissues were excised, and frozen immediately in liquid nitrogen, then stored at −80 °C until use. The experimental procedures were performed according to the standards of the Animal Care Policy of YFI.

### 2.3. Full-Length cDNA Cloning of PHD Genes

Full-length cDNAs of PHD genes were obtained by using reverse transcription-polymerase chain reaction (RT-PCR) and rapid amplification of cDNA ends (RACE). First, total RNA was isolated from the liver tissue of silver carp according to the instructions of Trizol reagent. Then, the concentration of the extracted RNA was detected by ultra-micro spectrophotometer (NP80, IMPLEN, München, Germany) and the integrity of RNA was detected by 1.5% agarose gel electrophoresis. Finally, first-strand cDNA was reverse-transcribed from the total RNA using HiScript^®^ Ⅲ 1st Strand cDNA Synthesis Kit (+gDNA wiper) (Vazyme, Nanjing, China) according to the manufacturer’s instructions. Primers (PHD1-P-F/R, PHD2-P-F/R, and PHD3-P-F/R) based on the predicted sequences of silver carp from our full-length transcriptome data (unpublished) were designed (Table 1). The PCR reactions were performed by denaturation at 98 °C for 2 min, followed by 35 cycles of denaturation at 98 °C for 10 s, annealing at 65 °C for 10 s, and elongation at 72 °C for 10 s; with an additional elongation at 72 °C for 2 min after the last cycle. The PCR products were detected by 1.5% agarose gel electrophoresis and sequenced by the Wuhan Tianyihuiyuan Biotech Company (Wuhan, China).

Subsequently, 5′ and 3′ RACE were performed using SMARTer RACE 5′/3′ kit (Takara, Dalian, China) according to the manufacturer’s protocol. The nested-PCR technique was employed with the gene-specific primers (GSP5/3-1 and GSP5/3-2) and universal primer (UPM or UPM short). The primers were designed using Primer Premier 5.0 software, and the sequences of the primers are listed in Table 1. The nested-PCR thermocycling conditions were as follows: 94 °C for 5 min, followed by 25 cycles at 94 °C for 30 s, 68 °C for 45 s, 72 °C for 3 min. In both 5′ and 3′ RACE, the first-strand cDNA was used as the template of the primary amplification, and the diluted primary PCR product was used as the template of the second amplification. 5′/3′-RACE PCR was carried out in a reaction volume as follows: 25 µL 2× SeqAmp buffer, 1 µL SeqAmp DNA polymerase, 2.5 µL cDNA template, 1 µL gene-specific primer (10 µmol/L), and 5 µL 10× Universal Primer A Mix (UPM). Deionized water was then added to achieve a total of 50 µL reaction volume. The PCR products were resolved using electrophoresis on a 1.5% agarose gel and purified using a gel extraction kit (D2500-02, Omega Bio-Tek, Norcross, GA, USA). Next, the purified products were ligated to vector pMD18T, and the recombinant plasmids were then transformed into competent *Escherichia coli* DH5α cells. Positive clones were screened and sequenced by the Wuhan Tianyihuiyuan Biotech Company.

### 2.4. Sequence and Evolutionary Analysis

The cDNA sequences of PHD genes were obtained by assembling the forward and reverse sequencing reads using the DNAMAN software. The online software ORF Finder (https://www.ncbi.nlm.nih.gov/orffinder/, accessed on 31 March 2021) was used to identify the open reading frame (ORF) in the sequence. The ORFs were translated into amino acid sequence and their basic physicochemical properties were predicted using ExPASy-ProtParam tool (https://web.expasy.org/protparam/, accessed on 1 April 2021). Then the online software SMART (http://smart.embl-heidelberg.de, accessed on 3 April 2021) and NCBI online CDD (https://www.ncbi.nlm.nih.gov/Structure/cdd/wrpsb.cgi, accessed on 3 April 2021) were used to analyze the domains in the deduced protein sequences. Alignments of deduced amino acid sequences were carried out using the ClustalW program. Gaps were removed manually. A Neighbor-Joining (NJ) phylogenetic tree was constructed with 1000 bootstrap replicates using MEGA X (Mega Limited, AKL, New Zealand). The evolutionary distances were computed using the JTT matrix-based method and in the units of the number of amino acid substitutions per site. The rate variation among sites was modeled with a gamma distribution (shape parameter = 1). This analysis involved 40 amino acid sequences. All ambiguous positions were removed for each sequence pair (pairwise deletion option). There were a total of 243 positions in the final dataset. Moreover, the maximum-likelihood (ML) tree was constructed through IQ-TREE version 1.5.5 [26] under the best-fitting model as estimated by ModelFinder implemented in IQTree with 1000 bootstrap replicates.

### 2.5. Reverse Transcription-Quantitative Polymerase Chain Reaction (RT-qPCR)

The expression patterns of PHD genes and the transcriptional responses of PHD genes to hypoxia were investigated using reverse transcription-quantitative polymerase chain reaction (RT-qPCR). Total RNAs were isolated from each tissue of the silver carp using Trizol reagent. After DNase treatment, 1000 ng of total RNA was reverse-transcribed to single-strand cDNA using a HiScript^®^ Ⅲ 1st Strand cDNA Synthesis Kit (+gDNA wiper) according to the manufacturer’s instructions. The primer pairs PHD1-Y- F/R for *HmPHD1*, PHD2-Y- F/R for *HmPHD2,* and PHD3-Y- F/R for *HmPHD3* were used (Table 1). Before RT-qPCR analysis, the standard curves for primer pair of *HmPHD1*, *HmPHD2,* and *HmPHD3* were generated by regression of Cq values and a series of ten-fold cDNA dilutions. Primer amplification efficiency was calculated from the slope of the corresponding standard curve, and the efficiency of PHD1-Y-F/R, PHD2-Y-F/R, and PHD3-Y-F/R was 97.63%, 92.49%, and 98.93%, respectively. The hypoxic-stable reference gene *β-actin* was used as the control [25] (Table 1). The RT-qPCR was performed using the ChamQTM Universal SYBR^®^ qPCR Master Mix (GeneBio Systems, Canada) with the following thermal cycling conditions: 95 °C for 30 s, 40 cycles of 95 °C for 10 s, and 60 °C for 30 s. Each experiment was performed independently for three times. The relative expression levels of PHD genes were normalized to that of *β-actin* quantification using the 2^−^^△△Ct^ method.

### 2.6. Statistical Analysis

The qRT-PCR data were expressed as mean ± SE. Statistical analysis (*t*-test) was performed using SPSS version 24.0 (SPSS Inc., Chicago, IL, USA). The level of statistical significance was set at *p* < 0.05 for all analyses.

## 3. Results

### 3.1. Molecular Characterization of PHD cDNAs in H. molitrix

Three full-length *HmPHD* cDNAs were obtained (Table S1). The *HmPHD1* cDNA comprised 2981 base pair (bp) in length, contained a 225 bp 5′ untranslated region (UTR), a 1449 bp ORF encoding a polypeptide of 482 amino acid (aa) residues, and a 1307 bp 3′ UTR including a putative polyadenylation signal (AATAAA) and a poly (A) tail. The *HmPHD2* cDNA consisted of a 150 bp 5′ UTR, a 1080 bp ORF encoding a polypeptide of 359 aa residues, and a 724 bp 3′ UTR. The *HmPHD3* cDNA contained a 75 bp of 5′ UTR, a 738 bp of ORF encoding a polypeptide of 245 aa residues, and a 1307 bp 3′ UTR. The full-length cDNA sequences and corresponding predicted protein sequences of PHD genes of *H. molitrix* are shown in Figures S1–S3.

**Figure 1:**
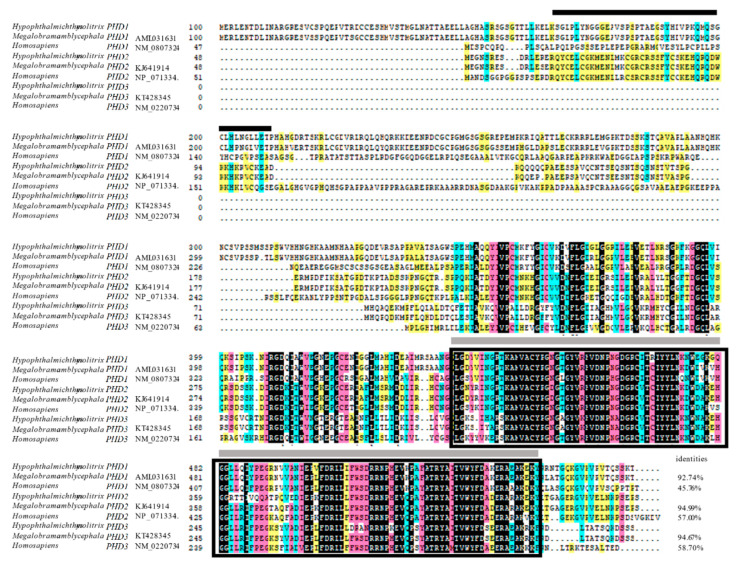
Alignment of *H. molitrix* PHDs deduced aa sequences with *M. amblycephala* and *Homo sapiens*. The GenBank accession numbers are indicated on the left of the alignment. The MYND-type zinc finger domain is included as a black straight line. The P4Hc homology region is included as an open box. The 2OG-Fe dioxygenase domain is included as a gray straight line.

The amino acid sequences of *H. molitrix* PHD proteins were highly similar to those of other species at the C-terminus, with typical P4HC (prolyl 4-hydroxylase alpha subunit homolog) domains in HmPHD1 (amino acid position 263–451), HmPHD2 (amino acid position 141–327), and HmPHD3 (amino acid position 34–220), which is the characteristic of the Fe (II) and 2-oxoglutarate-dependent dioxygenase family. The HmPHD2 protein also contained the typical motifs, MYND (myeloid, Nervy, and DEAF-1) type zinc finger interaction domain (Figure 1). The nucleotide sequence of PHD1, PHD2, and PHD3 of *H. molitrix* shared 92.74%, 94.99%, and 94.67% identity with their counterparts in *M. amblycephala*, and 45.76%, 57%, and 58.70% with their counterparts in *Homo sapiens*, respectively.

These results showed that HmPHD1 had the longest sequence length, while HmPHD3 had the shortest sequence length with the loss of amino acids occurring in its N-terminal part. C-terminal conserved domain was spared in all three proteins, while HmPHD2 also retained a unique N-terminal domain, indicating that these three proteins shared similar functions and HmPHD2 also had distinctive functions.

### 3.2. Evolutionary Relationships of PHD Genes

**Figure 2:**
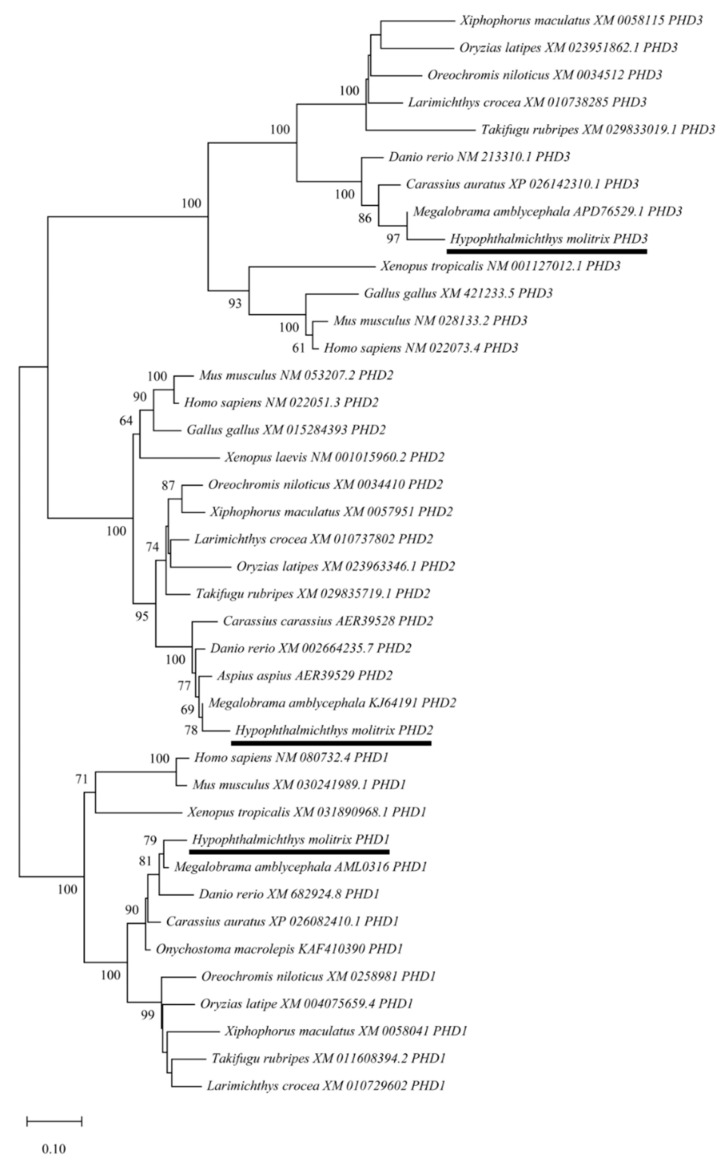
The phylogenetic tree of PHD genes constructed using neighbor-joining method in MEGA X. The bootstrap values mentioned at nodes were derived after 1000 replications. Each sequence is the name of the species and its GenBank accession number. PHDs of *H. molitrix* are marked by line.

To further clarify the evolutionary relationships of PHD genes among various species, a phylogenetic tree was constructed using the NJ method based on the putative amino acid sequences from different vertebrate species, including *H. molitrix*. The PHD genes were classified into three big clusters, and *HmPHD2* and *HmPHD3* were more closely related as they belonged to sister clades, whereas the clade of *HmPHD1* was relatively distant from these two. Additionally, the PHD genes of *H. molitrix* and *M. amblycephala* were clustered into a same clade, indicating that *H. molitrix* and *M. amblycephala* had a closer evolutionary relationship. Moreover, the phylogenetic tree showed that PHD genes of *H. molitrix* were most closely related to the genes from other cyprinids (Figure 2). Furthermore, the maximum-likelihood (ML) tree constructed through IQ-TREE is presented in Figure S4. The two phylogenetic trees were identical, thus the results were consistent.

### 3.3. Transcriptional Expression Patterns of PHD Genes in H. molitrix

**Figure 3:**
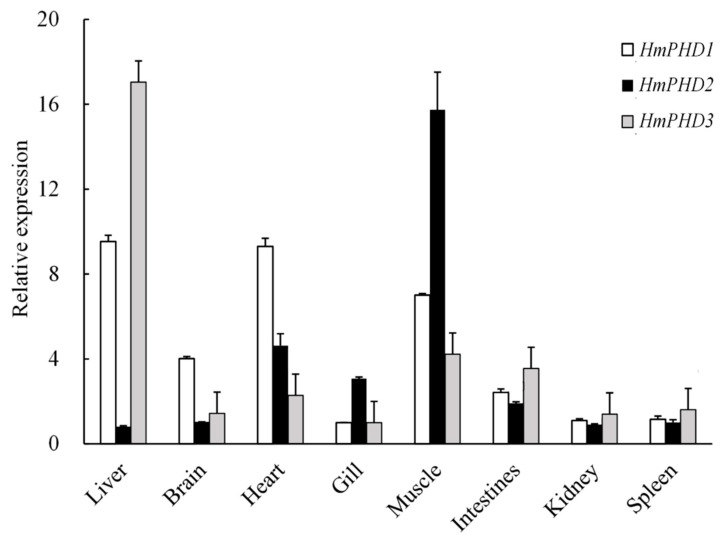
Tissue distributions of the transcripts of PHD genes in *H. molitrix* with *β-actin* as the positive control. Data were shown as mean ± SE (*n* = 3).

The transcripts of PHD genes were detected in all sampled tissues from *H. molitrix*. As shown in Figure 3, the high expression level of *HmPHD1* was found in liver and heart, followed by those in muscle and brain, while relatively low expression was detected in intestine, gill, kidney, and spleen. The *HmPHD2* mRNA was abundantly expressed in muscle, followed by that in heart, gill, and intestines. The *HmPHD3* mRNA had the highest expression level in liver. When compared with that of *HmPHD1* and *HmPHD3*, the mRNA level of *HmPHD2* was higher in gill and muscle, and was lower in liver, brain, intestines, kidney, and spleen, hence in contrast to *HmPHD2*, *HmPHD1,* and *HmPHD3* exhibited similar tissue distributions (Figure 3).

### 3.4. Transcriptional Response of PHD Genes in H. molitrix to Acute Hypoxia

**Figure 4:**
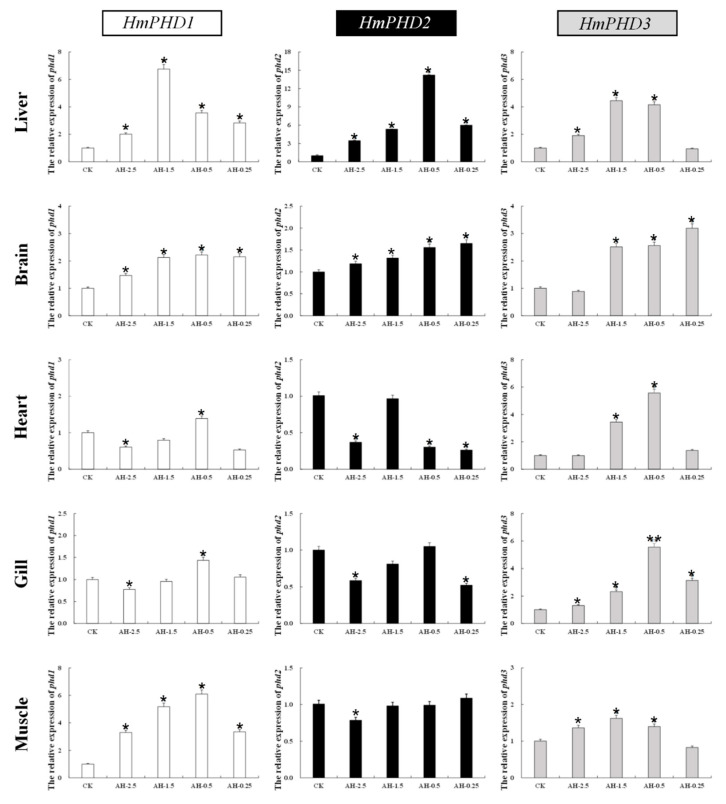
Relative expression levels of PHD genes in the liver, brain, heart, gill, and muscle after normoxia (CK), 0.5 h of hypoxia at 2.5 mg/L (AH-2.5), 1.5 mg/L (AH-1.5), 0.5 mg/L (AH-0.5), and 0.25 mg/L (AH-0.25), respectively. RT-qPCR data presented as mean ± SE. Significant differences at *p* < 0.05 are labeled with “*”, and *p* < 0.01 are labeled with “**”. AH: acute hypoxia.

The relative expression of PHD genes in different tissues (liver, brain, heart, gill, and muscle) of *H. molitrix* was analyzed under acute hypoxia stress. As shown in Figure 4, the mRNA levels of PHD genes increased significantly mainly in liver and brain (*p* < 0.05) under different low oxygen concentrations. The expression of *HmPHD1* and *HmPHD2* in heart and gill decreased significantly at DO of 2.5 mg/L (*p* < 0.05), while the expression of *HmPHD3* was elevated in heart and gill under acute hypoxia. Moreover, the mRNA levels of *HmPHD1* and *HmPHD3* in muscle increased significantly under the DO of 2.5 mg/L, 1.5 mg/L, and 0.5 mg/L (*p* < 0.05), while *HmPHD2* expression in muscle decreased significantly at DO of 2.5 mg/L compared with that under normoxic condition (*p* < 0.05).

These results showed that, under acute hypoxia for 0.5 h, all three PHD genes were induced in liver and brain, which indicated that brain and liver were the major organs for PHD genes to exert their function in *H. molitrix*. Different from *HmPHD1* and *HmPHD2*, *HmPHD3* showed a similar expression trend in all tissues, suggesting *HmPHD3* played roles with no overt tissue specificity under acute hypoxia conditions. Furthermore, among four different oxygen concentrations, PHD genes had the most extensive responses in DO of 1.5 mg/L and 0.5 mg/L, implying that these two DO were the best-fitted to study the transcriptional response of PHD genes to acute hypoxia in *H. molitrix*.

### 3.5. Transcriptional Response of PHD Genes to Continued Hypoxia and Reoxygenation in H. molitrix

**Figure 5:**
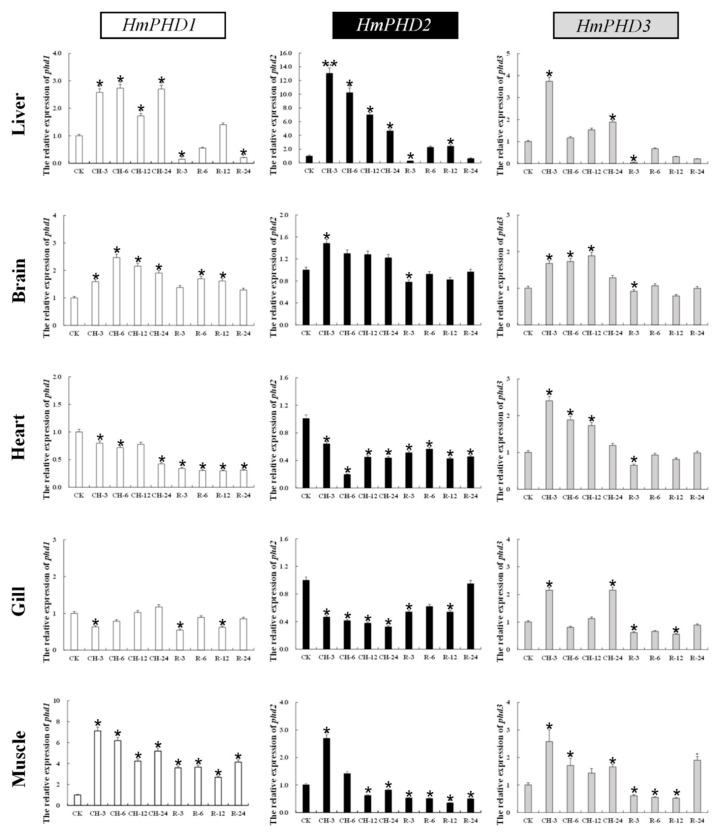
Relative expression levels of PHD genes in the liver, brain, heart, gills, and muscle after normoxia (CK), continued hypoxic at 3 h (CH-3), 6 h (CH-6), 12 h (CH-12), and 24 h (CH-24), and reoxygenation at 3 h (R-3), 6 h (R-6), 12 h (R-12), and 24 h (R-24). Values are shown as mean ± SE (*n* = 3). Significant differences at *p* < 0.05 are labeled with “*”, and *p* < 0.01 are labeled with “**”. CH: continued hypoxia (DO; 1.5 ± 0.3 mg/L). R: reoxygenation (DO; 6.5 ± 0.3 mg/L).

The expression patterns of *H. molitrix* PHD genes in response to continuous hypoxic stress and reoxygenation were further investigated. As shown in Figure 5, the mRNA levels of *HmPHD1*, *HmPHD2*, and *HmPHD3* in liver and brain increased significantly compared with those under normoxia after 3 h of hypoxia (*p* < 0.05) and remained relatively high until 24 h of hypoxic stress. The mRNA levels of *HmPHD1* and *HmPHD2* in heart and gill decreased significantly under 3 h of hypoxia stress (*p* < 0.05), while the expression of *HmPHD3* was increased significantly compared with normoxia (*p* < 0.05). The mNRA levels of *HmPHD1*, *HmPHD2,* and *HmPHD3* in muscle were increased significantly at 3 h under hypoxic stress (*p* < 0.05), and then decreased with time. Under hypoxic stress for 12 h and 24 h, the mRNA levels of *HmPHD1* and *HmPHD3* in muscle were still significantly higher than that under normoxic conditions (*p* < 0.05), while the expression level of *HmPHD2* was significantly lower than that under normoxic conditions. After 3 h of reoxygenation, the *HmPHD2* and *HmPHD3* expression levels were lower than those under normoxia in the detected tissues (liver, brain, heart, gill, and muscle). After reoxygenation for 24 h, the expression levels of *HmPHD1*, *HmPHD2,* and *HmPHD3* in gills all returned to their levels under normoxic conditions (*p* > 0.05).

These results showed that, in addition to liver and brain, muscle is a main organ that involved continued hypoxia stress. Except for *HmPHD1* and *HmPHD2* in heart and brain, three genes exhibited an obviously rapid but gradually attenuated response to continued hypoxic stress, implying that PHD genes were mainly involved in the early regulation of response to low-oxygen stress. Upon reoxygenation, the transcriptional expression of PHD genes showed a decreasing trend in most tissues, confirming that PHD genes were sensitive to oxygen in *H. molitrix*.

## 4. Discussion

Hypoxic conditions often occurs in aquaculture settings. In high-density fish farming, the intensity and duration of hypoxia depend on aeration capacity, fish biomass, feeding rate, etc. Changes in temperature promote high carbon dioxide emissions, and subsequently increased episodes of local acute hypoxia in the wild and in aquaculture ponds [27]. Silver carp, living in the upper water body and feeding on phytoplankton, are sensitive to variation in the DO content. A sudden lack of oxygen will result in mortality among the fish, reducing algae availability and triggering water pollution, and eventually leading to pond turnover [28]. Thus, it is necessary to study the responses and adaptive mechanisms to hypoxia challenge in silver carp.

In the present study, the full-length cDNA sequences of *H. molitrix* PHD genes were obtained. Multiple alignment showed that these encoded proteins belonged to the Fe (II) and 2-oxoglutarate-dependent dioxygenase superfamily and were composed of similar functional domains, i.e., the 2-oxoglutarate (2OG)-Fe (II) oxygenase superfamily domain and P4Hc (prolyl 4-hydroxylase alpha subunit homologues) domain. The 2OG-Fe (II) oxygenase superfamily domain is a characteristic domain of PHDs, which is important in the regulation of hypoxia inducible transcription factors [29]. The P4Hc domain is the hydroxylation functional domain of PHD, which catalyzes the proline hydroxylation of collagen to form 4-hydroxyproline [30]. Furthermore, it is the domain responsible for the hydroxylation of HIF-1α as a regulatory factor of the hypoxia response [31,32]. Therefore, these conserved domains indicate that the PHD1, PHD2, and PHD3 proteins of *H. molitrix* have similar functions, and the same conserved domains are found in PHDs of other species, such as *Mus musculus* and *X. laevis* [33,34]. However, the MYND zinc finger domain in the N-terminal of HmPHD2 was the main difference between PHD proteins in *H. molitrix* [35,36], and this domain is unique to PHD2 proteins among the PHD family in vertebrates. This MYND zinc finger domain can anchor the N-terminus of PHD2 to mitochondria or the endoplasmic reticulum membrane via the protein FKBP prolyl isomerase 8 (FKBP38) [37]. Among the PHD homologous family in metazoans, PHD2 represents the most primitive form according to its conserved amino acids and its domain structure [32,36,38]. Phylogenetic analysis showed that PHD1 proteins, PHD2 proteins, and PHD3 proteins in different fish clustered into a large clade respectively, indicating that PHD were relatively conservative in evolution. The phylogenetic tree analysis also showed that PHD2 and PHD3 are more closely related to each other than they are to PHD1, which suggested that PHD2 and PHD3 may have similar functions [39].

The *PHD* mRNAs were expressed but with different levels in various tissues of *H. molitrix* under normoxic condition. The *HmPHD1* expression level was the highest in liver and heart, which was similar to the results that *PHD1* mRNA is highly expressed in the testis, followed by the liver, heart, brain, and kidney in human [40]. The *HmPHD2* mRNA was highly expressed in muscle, heart, gill, and intestine, while the *HmPHD3* mRNA was highly expressed in liver, heart, muscle, and intestine. This was consistent with the results from *M. amblycephala*, in which the expression level of *MaPHD2* was the highest in muscle [23] and lower in other tissues, while *MaPHD3* [24] was highly expressed in the liver. However, our data were inconsistent with the results that *PHD2* and *PHD3* had the highest expression in heart, followed by brain and kidney, in mammals [40]. In addition, *HmPHD2* expression was complementary to that of *HmPHD3* in various tissues. This was consistent with studies in *Xenopus laevis*. *XlPHD2* was mainly expressed in the eyes, brain, and intestinal tract, but showed low expression in the liver, pancreas, and kidney; *XlPHD3* was highly expressed in the liver, pancreas, and kidney, but showed lower expression in the eyes, brain, and intestinal tract [35]. In mammals, each PHD shows a different hydroxylation preference for HIF-αs [41]. Specific silencing of PHD2 with short interfering RNAs proved that PHD2 should be the main cellular oxygen sensor setting the low steady-state levels of HIF-1α in normoxia [38]. However, the PHD1/PHD3 double knockout mouse showed preferential HIF-2α rather than HIF-1α stabilization in liver [42]. Loss of PHD1 catalytic activity in breast cancer cells inhibits estrogen-dependent tumorigenesis, and PHD1 depletion also impairs the fitness of lung, brain, and hematopoietic cancer lines [43].

Under acute hypoxic stress, the *HmPHD1* was upregulated significantly in the liver, brain, and muscle, but downregulated significantly in heart and gill. The expression of *MaPHD1* in the heart also decreased after hypoxia treatment [23]. In a study of *Sillago sihama*, the expression of the *SsPHD1* in heart and gills increased significantly after hypoxic treatment [22]. This might be caused by the different intensity and duration of hypoxic treatment or the species-specific expression patterns under hypoxic conditions. The *HmPHD2* and *HmPHD3* showed the same changing trend in the liver and brain under acute hypoxia stress, and their expression levels were significantly increased. This was consistent with the results on *M. amblycephala*. During hypoxic exposure, the expression levels of *MaPHD2* [21] and *MaPHD3* [24], were significantly increased in the liver and brain. In the heart, gill, and muscle, the expression of *HmPHD2* decreased significantly, while the expression of *HmPHD3* increased significantly. This is also consistent with the results on the cyprinid fish *M. amblycephala*. In *M. amblycephala*, the expression level of *MaPHD2* [21] was decreased in blood, head, kidney, muscle, and gill after hypoxia stress, while the expression of *MaPHD3* [24] increased significantly in the blood, liver, spleen, muscle, brain, gill, heart, intestine, and kidney tissues. However, in the non-carp species *S. sihama*, the expression of *SsPHD2* increased significantly and the expression of *SsPHD3* decreased significantly in gill after hypoxic stress, contrary to the results of the present study [22]. The expression of *MaPHD3* showed no overt tissue specificity under acute hypoxia conditions, which might be possibly related to the HREs in its promoter potently bonded by HIF-1α [24]. This suggested that PHD genes play similar but not exactly the same roles in response to hypoxic stress in different fish. In closely related fish (such as cyprinids), PHD genes are likely to play more consistent roles in the same tissues.

Under continued hypoxia stress, PHD activity is repressed by insufficient oxygen concentrations, and the variation in the levels of transcripts of PHD genes might be induced to relieve this repression. However, organisms adapted to a hypoxic environment over time accompanied with the decrease of PHD genes expression. The expression levels of PHD genes in the detected tissues were lower than normal after 3 h of reoxygenation, which might reflect the recovery of hydroxylation of HIF-1α by PHDs as oxygen receptors after the recovery of oxygen, and the elimination of a large amount of accumulated HIF-1α, such that the expression levels of PHD genes decreased. After 24 h of reoxygenation, the expression of PHD genes in gill returned to the same level as normaxia, which suggested the organizational superiority of the gill in coping with hypoxia stress compared with that of other tissues. Gill was directly exposed to water throughout the experiment [44] and was likely to be the earliest tissue to detect the DO decrease under hypoxia, such that the PHD genes in gill returned to normal levels after reoxygenation faster than they did in the other organs. The brain and heart, playing important roles in the survival of the organism, were protected by the redistribution of oxygen in the blood. The expression levels of *HmPHD2* and *HmPHD3* in the brain were restored to normoxic levels after reoxygenation. Similar results have also been reported in *Pelteobagrus vachelli*, which were inferred from the blood flow rearrangement in the brain during hypoxia challenge [45]. However, after 24 h of reoxygenation, the expression levels of *HmPHD1* in liver, brain, heart, and muscle; *2* in muscle and heart; and *HmPHD3* in muscle and liver still had not recovered to normoxic levels. In the study on *S. sihama*, the expression levels of *SsPHD2* and *SsPHD3* returned to normoxic levels in gill and heart after reoxygenation for 24 h [22]. This might be caused by the different conditioning treatments. In the present study, *H. molitrix* were exposed to reoxygenate after 24 h of hypoxia, while in the study of *S. sihama* [22], the reoxygenation was performed after 4 h of hypoxia, indicating that continuous hypoxic stress would cause serious damage to the heart tissue, such that a recovery period of 24 h was not sufficient to allow the expression levels of the *HmPHD1*, *HmPHD2*, and *HmPHD3* to fully recover.

In the present study, because of the limitations of our detection conditions, we mainly focus on the changes of PHD genes in the levels of transcripts. However, evaluating the activity of the PHDs in the different tissues at different times were meaningful, and we will try to master the technique to assess the activity of PHDs in the further studies.

## 5. Conclusions

Three full-length *PHD* cDNAs were obtained from *H. molitrix* by RACE. The amino acid sequences of PHD1, PHD2, and PHD3 from *H. molitrix* were highly similar to those of other species at the C-terminus with typical P4HC domains. The evolutionary relationships of PHD genes among various species were clarified with a phylogenetic tree, and *HmPHD2* and *HmPHD3* were more closely related. The transcripts of PHD genes are ubiquitously distributed in *H. molitrix* tissues, with the highest expressional level of *HmPHD1* and *HmPHD3* in liver, and *HmPHD2* in muscle. Under acute hypoxic condition, PHD genes showed a tendency of increased expression mainly in the liver and brain. Meanwhile, under continued hypoxic condition, the expression of PHD genes was up-regulated significantly after hypoxic treatment for 3 h, and then maintained their relatively high expression level up to 24 h, and upon reoxygenation, the transcriptional expression of PHD genes showed a decreasing trend in most tissues. This study is of great relevance for advancing our understanding of how PHDs perform regulation functions at the transcriptional level when addressing the hypoxic challenge and provides a reference for the subsequent research of the molecular mechanisms underlying hypoxia adaptation in silver carp.

## Figures and Tables

**Table 1:** Primers used for PCR.

Primer Names	Primer Sequences (5′–3′)	Application
PHD1-P-F	GCTTTTGAAGGAGTTGAAGAGTGGGT	CDS amplification
PHD1-P-R	GTCCGTCGCCGTTAGGATTGTC	
PHD2-P-F	GGGAAAATGGAGAACCTGATGAAGTG	
PHD2-P-R	TTGTGGCATAGGCTGGCTGGAC	
PHD3-P-F	CCGAGGCTACTTTTACGTGGATAATTT	
PHD3-P-R	GCATACCTTGTAGCATAGGACGGTTG	
UPM short	CTAATACGACTCACTATAGGGC	5′ and 3′ RACE
UPM	CTAATACGACTCACTATAGGGCAAGCAGTGGTATCAACGCAGAGT	
PHD1-GSP5-1	ACCCATTTCAAGCCTCCTCCTTTT	5′ RACE
PHD1-GSP5-2	AAGTCCTGTCCCCGTGAGCATG	
PHD2-GSP5-1	GCACGTCCTCCAGAATACTGCGTC	
PHD2-GSP5-2	CACCCGCTTGTGCTTCTTCCAG	
PHD3-GSP5-1	ATTTTATCCCCTCTGATGTTTGTCCTG	
PHD3-GSP5-2	CCGTCGTTGAGAATCCCACAGTAA	
PHD1-GSP3-1	TGGACAATCCTAACGGCGACG	3′ RACE
PHD1-GSP3-2	TGGGAGGGGAAGGGGCAGGGAGGTT	
PHD2-GSP3-1	ATAACCCTAACGGAGATGG	
PHD2-GSP3-2	CATTGAGCCCAAGTTTGA	
PHD3-GSP3-1	GTGTGGGTCAACTGGGCAAAAGCAT	
PHD3-GSP3-2	GCCGCTGCGTCACCTGTAT	
PHD1-Y-F	TCGGAAATGCCTAATGGACTG	RT-qPCR
PHD1-Y-R	GCTTTGTGCCCATTGTGATG	
PHD2-Y- F	CGACACTGTAACGGGAAACTGG	
PHD2-Y-R	TCTGGAAAGATCCGCAAAAGG	
PHD3-Y- F	AGTTCGAGACTTTGGCTGTC	
PHD3-Y-R	CCCCTCTGATGTTTGTCCTG	
*β-actin* F	GAACCCCAAAGCCAACAG	
*β-actin* R	CAGAGTCCATCACGATACCAG	

## Data Availability

The data presented in this study are available in the article.

## Supplementary Materials

The following are available online at https://www.mdpi.com/article/10.3390/ani12020131/s1. Figure S1: Full length cDNA sequence and protein sequence of *HmPHD1*. Figure S2: Full length cDNA sequence and protein sequence of *HmPHD2.* Figure S3: Full length cDNA sequence and protein sequence of *HmPHD3.* Figure S4: The maximum-likelihood (ML) tree constructed through IQ-TREE. Table S1: The sequence of full-length cDNA of three PHD genes in *Hypophthalmichthys molitrix*.

## Abbreviations

bp	Base pair
PHD	Prolyl hydroxylase domain
*HmPHD*	PHD genes of *H. molitrix*
HmPHD	PHD proteins of *H. molitrix*
ORFs	Open reading frames
DO	Dissolved oxygen
HIFs	Hypoxia-inducible factors
ODDD	Oxygen dependent degradation domain
VHL	Von Hippel-Lindau tumor suppressor protein
HRE	Hypoxia response element
YFI	Yangtze River Fisheries Research Institute
AH	Acute hypoxia
CK	Control group
RT-PCR	Reverse transcription-polymerase chain reaction
RACE	Rapid amplification of cDNA ends
aa	amino acid
