# Supplementary figures and images for: Molecular Characterization and Response of Prolyl Hydroxylase Domain (PHD) Genes to Hypoxia Stress in Hypophthalmichthys molitrix
Source: Animals (Basel). 2022 Jan 6;12(2):131. doi: 10.3390/ani12020131 (PMC8772553; doi:10.3390/ani12020131)

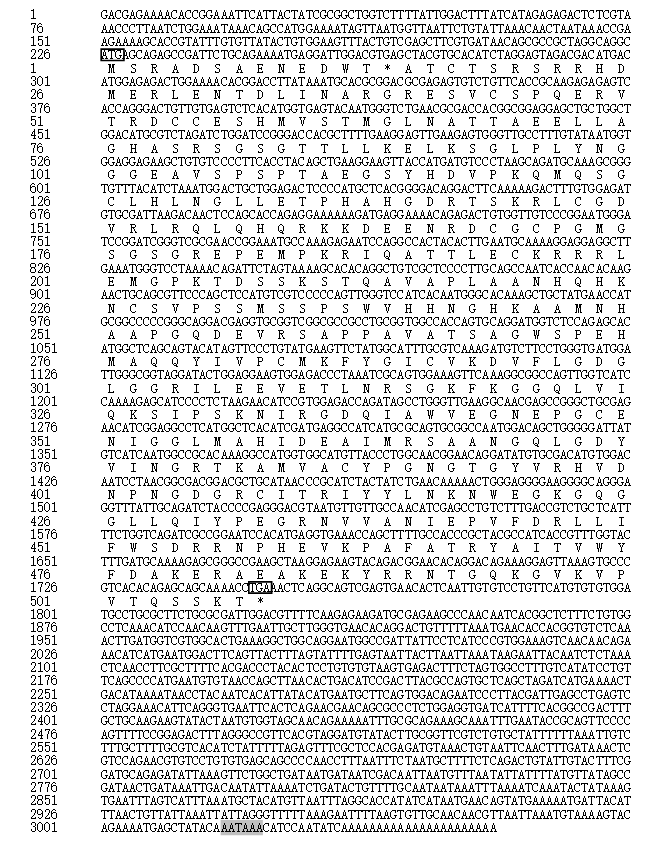

Supplement: Supplementary file 1 [file animals-12-00131-s001.zip › Figure S1.tif]

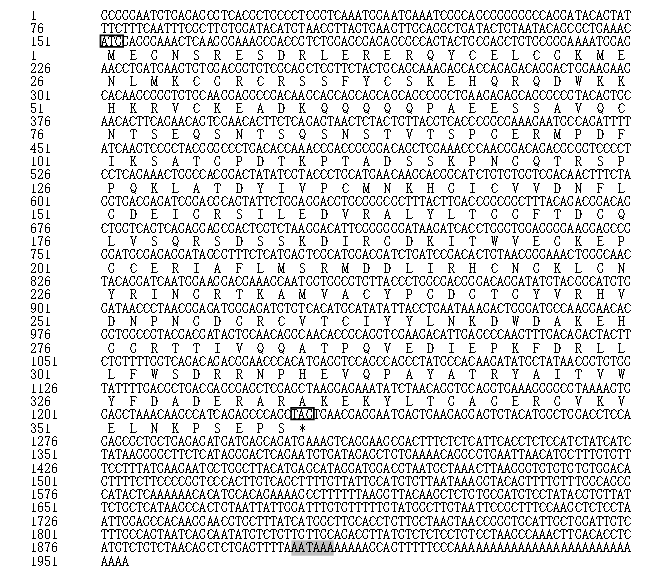

Supplement: Supplementary file 1 [file animals-12-00131-s001.zip › Figure S2.tif]

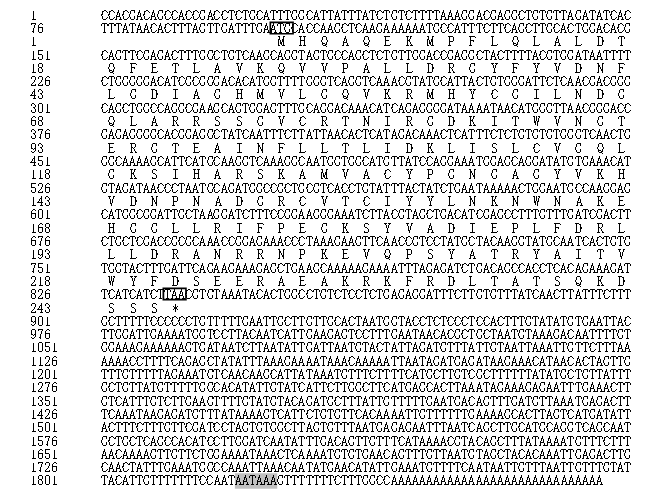

Supplement: Supplementary file 1 [file animals-12-00131-s001.zip › Figure S3.tif]

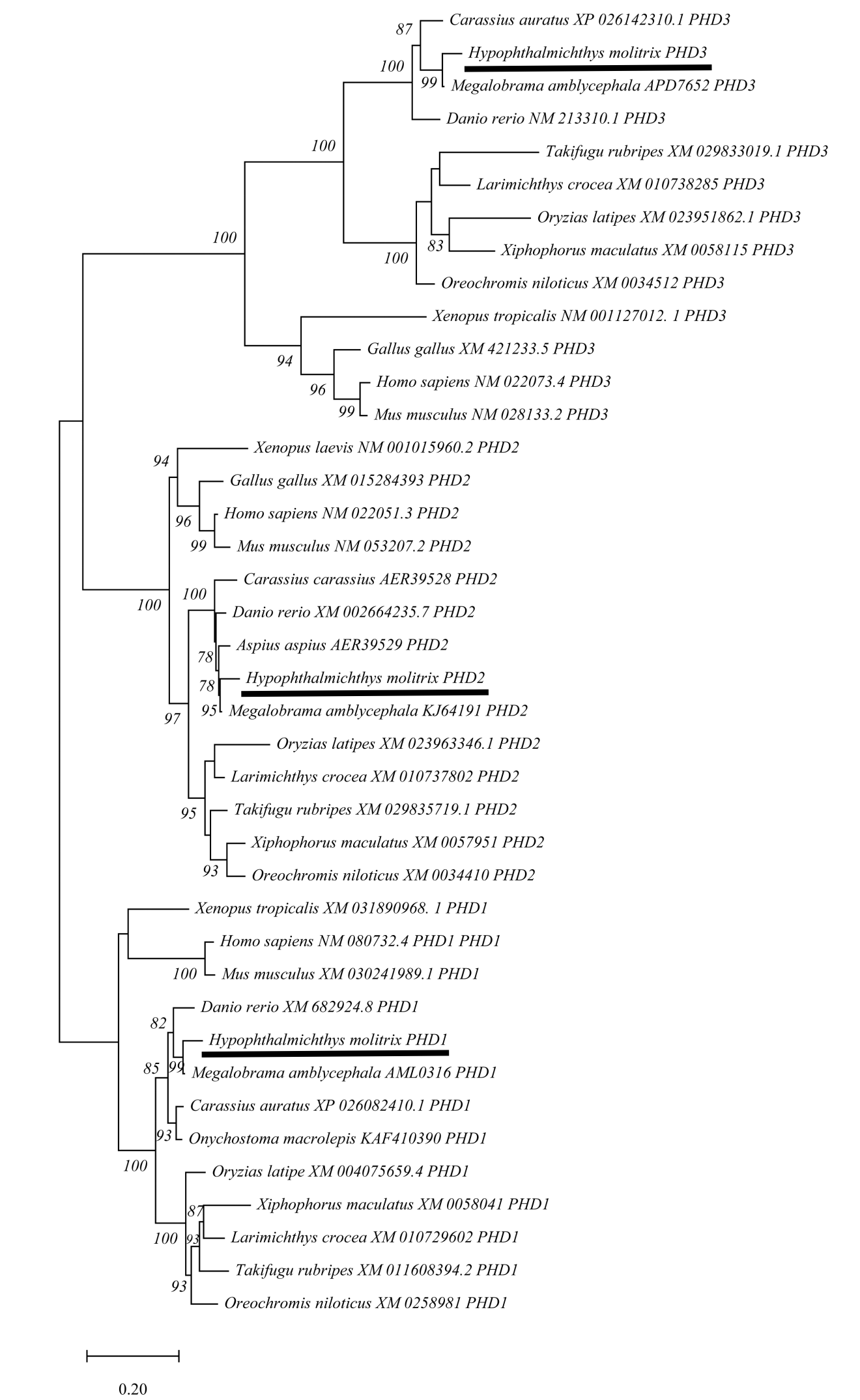

Supplement: Supplementary file 1 [file animals-12-00131-s001.zip › Figure S4.tif]
